# Lower limb hyperthermia augments functional hyperaemia during small muscle mass exercise similarly in trained elderly and young humans

**DOI:** 10.1113/EP091275

**Published:** 2023-07-06

**Authors:** Nuno Koch Esteves, Ashraf W. Khir, José González‐Alonso

**Affiliations:** ^1^ Division of Sport, Health, and Exercise Sciences, Department of Life Sciences Brunel University London Uxbridge UK; ^2^ Department of Engineering Durham University Durham UK

**Keywords:** ageing, blood flow, exercise, haemodynamics, heat

## Abstract

Heat and exercise therapies are recommended to improve vascular health across the lifespan. However, the haemodynamic effects of hyperthermia, exercise and their combination are inconsistent in young and elderly people. Here we investigated the acute effects of local‐limb hyperthermia and exercise on limb haemodynamics in nine healthy, trained elderly (69 ± 5 years) and 10 young (26 ± 7 years) adults, hypothesising that the combination of local hyperthermia and exercise interact to increase leg perfusion, albeit to a lesser extent in the elderly. Participants underwent 90 min of single whole‐leg heating, with the contralateral leg remaining as control, followed by 10 min of low‐intensity incremental single‐leg knee‐extensor exercise with both the heated and control legs. Temperature profiles and leg haemodynamics at the femoral and popliteal arteries were measured. In both groups, heating increased whole‐leg skin temperature and blood flow by 9.5 ± 1.2°C and 0.7 ± 0.2 L min^−1^ (>3‐fold), respectively (*P* < 0.0001). Blood flow in the heated leg remained 0.7 ± 0.6 and 1.0 ± 0.8 L min^−1^ higher during exercise at 6 and 12 W, respectively (*P* < 0.0001). However, there were no differences in limb haemodynamics between cohorts, other than the elderly group exhibiting a 16 ± 6% larger arterial diameter and a 51 ± 6% lower blood velocity following heating (*P* < 0.0001). In conclusion, local hyperthermia‐induced limb hyperperfusion and/or small muscle mass exercise hyperaemia are preserved in trained older people despite evident age‐related structural and functional alterations in their leg conduit arteries.

## Abstract (highlights)

- **What is the central question of the study?**
  Ageing is postulated to lead to underperfusion of human limb tissues during passive and exertional hyperthermia, but findings to date have been equivocal. Thus, does age have an independent adverse effect on local haemodynamics during passive single‐leg hyperthermia, single‐leg knee‐extensor exercise and their combination?
- **What is the main finding and its importance?**
  Local hyperthermia increased leg blood flow over three‐fold and had an additive effect during knee‐extensor exercise with no absolute differences in leg perfusion between the healthy, exercise‐trained elderly and the young groups. Our findings indicate that age per se does not compromise lower limb hyperaemia during local hyperthermia and/or small muscle mass exercise.

## 1 INTRODUCTION

Ageing is associated with numerous structural–functional alterations in the peripheral vasculature and the myocardium. These typically include increases in arterial diameter, wall thickness and stiffness, left ventricular hypertrophy, elevations in arterial blood pressure and reductions in maximal cardiac output (Ferrari et al., 2003; Hossack & Bruce, 1982; Nichols et al., 2022; Rosenthal, 1987; Sonesson et al., 1993; Thijssen et al., 2016). Maximal limb hyperaemia (functional hyperaemia) is also reduced in elderly populations during maximal aerobic exercise, a physical effort that engages a large muscle mass (Beere et al., 1999; Poole et al., 2003; Proctor et al., 2004, 1998; Wahren et al., 1974). Findings during small muscle mass knee‐extensor exercise are inconsistent, however, with studies showing a blunted hyperaemic response to low‐ to maximal‐intensity exercise in sedentary and normally active elderly participants (Donato et al., 2006; Lawrenson et al., 2003; Mortensen et al., 2012) but not in lifelong endurance‐trained elderly counterparts (Mortensen et al., 2012). The age‐related attenuation in functional hyperaemia during low‐intensity knee‐extensor exercise is surprising given that a number of studies have shown no differences in lower‐limb blood flow between young and aged adults during matched low‐intensity cycling exercise in sedentary (Beere et al., 1999; Poole et al., 2003), normally active (Proctor et al., 2003) and endurance‐trained (Wahren et al., 1974) participants. To address this discrepancy in the literature and minimise the influences of different levels of aerobic fitness and cardiovascular capacity, the impact of ageing on functional hyperaemia can be investigated in exercised‐trained elderly people during low‐intensity exercise.

Hyperthermia – which can also instigate profound increases in limb blood flow – may provide further insight into the age‐related alterations in vascular function. A wealth of literature exists on the blood flow responses to passive hyperthermia in young and aged subjects indicating that elderly adults have an attenuated hyperthermia‐induced elevation in forearm perfusion (Armstrong & Kenney, 1993; Minson et al., 1998; Rooke et al., 1994; Sagawa et al., 1988) and leg perfusion (Kenny et al., 2017; Romero et al., 2017). There are discrepancies in the literature, though, as two studies have reported comparable magnitude of hyperperfusion between aged cohorts in the human forearm (Kenny et al., 2017) and leg (Engelland et al., 2020). Moreover, the impact of superimposing local hyperthermia to exercise remains unresolved irrespective of the population. Three studies in young individuals show no effect of localised thigh heating during knee‐extensor exercise (Ferguson et al., 2006), whole‐body heating during prolonged submaximal single‐leg and two‐leg cycling (Savard et al., 1988), or incremental two‐leg cycling to exhaustion (Trangmar et al., 2017). Conversely, two recent investigations revealed an additive effect of whole‐body hyperthermia in young participants, with functional hyperaemia increasing by 0.6−0.7 L min^−1^ during single‐leg knee‐extensor exercise in comparison to normothermic conditions (Chiesa et al., 2015; Pearson et al., 2011). To our knowledge, the effect of lower‐limb hyperthermia on the magnitude of functional hyperaemia during small muscle mass exercise in elderly adults has never been investigated. Addressing this gap in knowledge is important in determining the potential of local heating and light exercise as a therapeutic intervention to improve health in people with reduced functional capacity.

The aim of the present study was to comprehensively examine and compare the haemodynamic responses to single‐leg hyperthermia, one‐legged knee‐extensor exercise and combined single‐leg hyperthermia and knee‐extensor exercise in aged and young adults. It was hypothesised that: (a) single‐leg hyperthermia and one‐legged knee‐extensor exercise would increase limb blood flow, which would be tightly coupled to increases in local limb temperature and/or metabolic demands; and (b) elderly participants would demonstrate an attenuated hyperaemic response to single‐leg hyperthermia and knee‐extensor exercise, in comparison to the young adult group.

## 2 METHODS

### 2.1 Ethical approval

The study was approved by the Brunel University London Research Ethics Committee (31692‐A‐Nov/2021‐34810‐2) and was performed in accordance with the *Declaration of Helsinki*, except for registration in a database. All participants provided informed written consent prior to their participation in the present study.

### 2.2 Participants

A group of nine healthy elderly adults (three women) and a group of 10 healthy young (three women) individuals participated in this study. The elderly adults had a mean ± SD age of 69 ± 5 years, a height of 172.9 ± 4.8 cm and body mass of 68.7 ± 11.7 kg, whereas the corresponding values for the young cohort were 26 ± 7 years, 171.7 ± 6.8 cm and 71.0 ± 9.7 kg (Table 1). Prior to the start of the study, informed written consent was obtained from all participants following a detailed written and verbal explanation of the experimental protocol. Participants were considered healthy and trained following the completion of a health questionnaire and a basic cardiovascular screening. All participants regularly engaged in structured sports, endurance and/or strength and conditioning training 3–7 times per week, with each session lasting 30–120 min. There were no differences in exercise frequency, duration and modalities between aged cohorts. Participants refrained from heavy exercise for 48 h, alcohol consumption for 24 h and caffeine consumption for 12 h before the commencement of the protocols. Moreover, young female participants, who had not undergone menopause, were requested to schedule their laboratory visit during the first 7 days follow menses – that is, the early follicular phase – as it is commonly associated with the lowest levels of oestrogen and progesterone.

**TABLE 1:** Participant demographic and anthropometric characteristics.

Variables	Elderly (*n* = 9)	Young (*n* = 10)
Age (years)	69 ± 5	26 ± 7
Sex, *n* (%)		
Female	3 (30%)	3 (30%)
Male	6 (60%)	7 (70%)
Height (cm)	172.9 ± 4.8	171.7 ± 6.8
Mass (kg)	68.7 ± 11.7	71.0 ± 9.7
Right leg volume (L)	10.2 ± 1.6	11.0 ± 1.9
Right leg lean volume (%)	76.3 ± 7.9	78.6 ± 6.7
Right leg non‐lean volume (%)	23.7 ± 7.9	21.4 ± 6.7
Left leg volume (L)	9.8 ± 1.9	11.3 ± 1.8
Left leg lean volume (%)	76.3 ± 8.1	78.7 ± 6.9
Left leg non‐lean volume (%)	23.7 ± 8.1	21.3 ± 6.9

Values are means ± SD for nine elderly and 10 young participants.

### 2.3 Experimental protocols

Participants were asked to consume their usual breakfast and report to the laboratory between 08.00 and 10.00 h. They were weighed in a semi‐nude state and had their height (SECA 798 Scale, SECA, Hamburg, Germany) and leg anthropometric measurements recorded. The latter data allowed an estimate of leg composition using the method reported by Jones and Pearson (1969). Seven leg circumferences were taken at the gluteal furrow, one‐third subischial (one‐third of the distance between the gluteal furrow and the popliteal crease), the minimum circumference above the knee, the maximum circumference at the knee joint, the minimum circumference below the knee, the maximum circumference at the calf and the minimum circumference at the ankle joint. Additionally, skinfold measurements were obtained at the following four sites: one‐third subischial (anterior and posterior sites) and at the maximum calf circumference (lateral and medial sites) using skinfold callipers (Jones & Pearson, 1969). Subsequently, participants sat in a semi‐recumbent position on the chair of a custom‐built knee‐extensor ergometer within the laboratory at an ambient temperature and humidity of 21°C and 30–40%, respectively. Participants were then instrumented with ECG electrodes and the finometer upper‐arm and middle finger cuffs to allow the assessment of central haemodynamics.

#### 2.3.1 Familiarisation protocol

**FIGURE 1:**
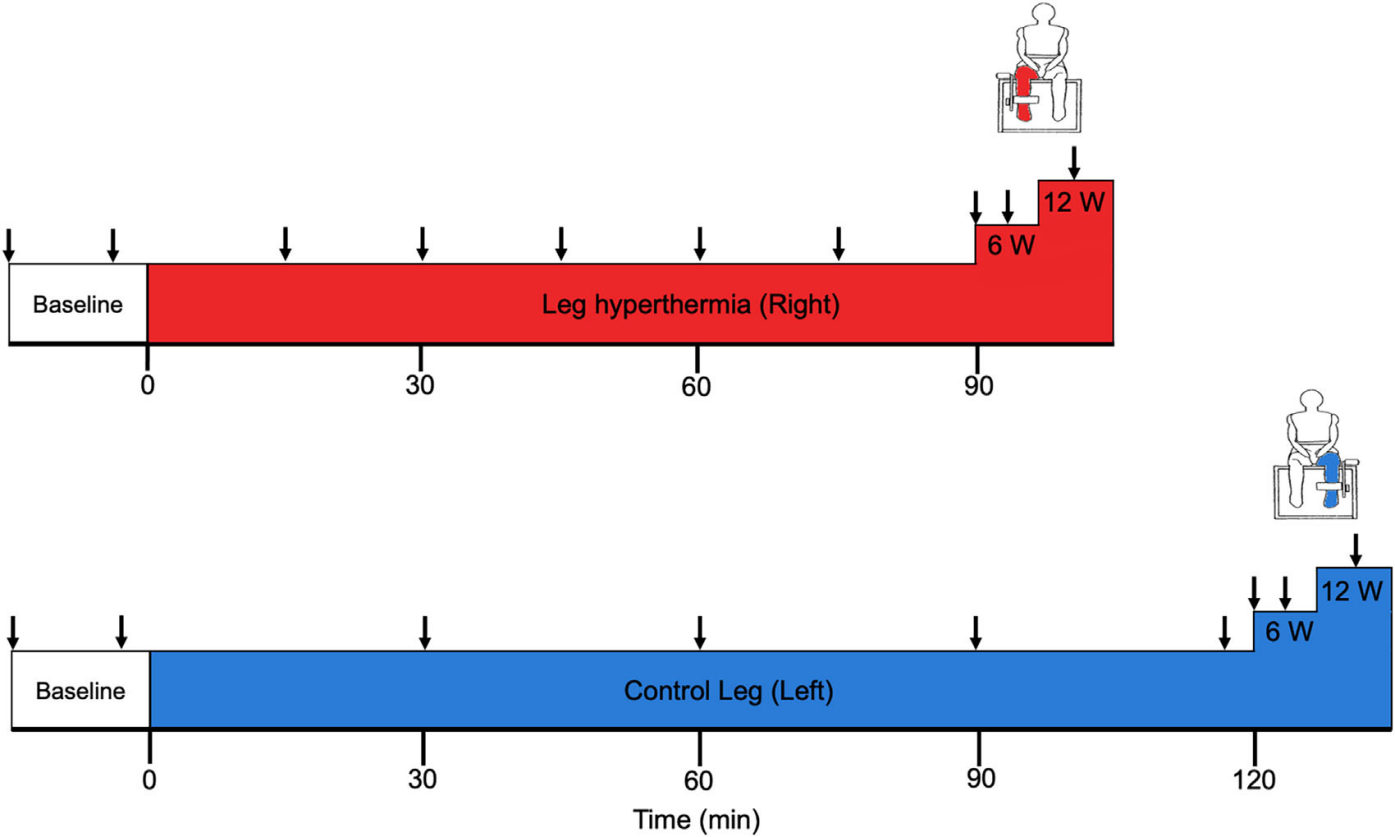
Schematic of experimental protocol. Downward arrows illustrate the times in which an ultrasound blood flow measurement was conducted. Blood flow was measured at the common, superficial and profunda femoral arteries and popliteal artery during rest and passive heating; however, blood flow was solely measured at the common and superficial femoral arteries during exercise. Core temperature, leg temperatures, leg tissue oxygen saturation and central haemodynamics were measured continuously throughout the protocol.

The familiarisation protocol commenced with a basic cardiovascular screening where participants had their ECG, cardiac output, stroke volume and leg blood flow at the common and superficial femoral arteries (SFAs) measured and evaluated. If participants reported abnormal values which were indicative of any underlying health issues, their participation in the study was terminated and they were recommended to schedule an appointment with their health practitioner. Following the successful completion of the health screening, participants had their left ankle and foot inserted and strapped into the boot of a modified dynamic knee‐extensor exercise Monark ergometer. Participants were familiarised with the one‐legged knee‐extensor exercise (Andersen & Saltin, 1985), exercising on an unloaded ergometer for 5 min (Figure 1). Once the familiarisation with their left leg was completed, the participants’ right ankle and foot were inserted and strapped into the boot of the ergometer, and the familiarisation protocol was repeated once more.

#### 2.3.2 Experimental protocol

The schematic for the experimental protocol is illustrated in Figure 1. Following the successful completion of the familiarisation protocol, participants rested for the following 30 min. During this time, they were instrumented with tissue oxygenation optode pads and temperature thermistors (described below). Once the participant was successfully instrumented and completed the 30‐min rest period, the experimental protocol was initiated with baseline measurements of the common femoral artery (CFA), SFA, profunda (deep) femoral artery (PFA) and popliteal artery (POA) in both the right and left legs. Next, participants were fitted with a custom‐made water‐perfusion trouser on their right leg, which was then wrapped in a survival blanket to optimise the heating procedure by limiting heat loss from the trouser to the surrounding environment. The trouser was connected to a thermostatically controlled water circulator (Julabo F‐34, Seelbach, Germany), which continuously circulated water at a temperature of 58°C (Figure 1). While the water temperature leaving the water circulator was substantially hotter than that commonly reported in the literature (∼48°C) – including other previous studies from our laboratory (Koch Esteves et al., 2021) – the actual skin temperature was similar to those in previous studies due to the larger heat loss through the smaller diameter tubing and thus reduced flow rate in the custom‐made water‐perfused trouser. During the 1.5‐h right leg heating protocol, blood flow was measured every 15 min at the CFA, SFA, PFA and POA in the heated leg, and every 30 min in the control (left) leg.

Following passive whole leg heating, participants performed incremental one‐legged knee‐extensor exercise. The one‐legged knee‐extensor exercise consisted of two 5‐min bouts, the first at 6 W and the second at 12 W (Figure 1). Intensity was controlled by increasing the resistance on the flywheel via metal weights. To account for the minor variations in cadence – that is, differences in the number of knee extensions per minute – individual work rates were calculated for each stage using the following formula: $\mathrm{Work}\;\mathrm{Rate}\;=\bar{\mathrm{Cadence}}\;\times \mathrm{Resistance}$. During the exercise protocol, blood flow was measured at the CFA and SFA of the right leg during the middle of each exercise bout – that is, at 2.5 and 7.5 min. Once the exercise protocol was completed, the heated trouser was removed, thus concluding the protocol for the right leg. Subsequently, the control left leg was inserted and strapped into the boot of the exercise ergometer. The exercise protocol for the left leg was the same to the right leg protocol, except for the fact that the heated water‐perfused trouser was not utilised. Baseline blood flow measures were taken at the CFA, SFA, PFA and POA of the left leg. This was succeeded by an incremental exercise protocol (6 and 12 W for 5 min each) with blood flow being assessed at the same time points and vessels as in the heat leg (Figure 1).

### 2.4 Temperature measurements

Core temperature (*T*_c_) was measured using a rectal probe (Ret‐1 Special, Physitemp, Clifton, NJ, USA) which was self‐inserted 15 cm past the sphincter muscle. Skin temperature (*T*_sk_) in the quadriceps, hamstrings and calf for both legs was measured using commercially available thermistors (IT‐18, Physitemp) which were securely held in place using medical tape. *T*_c_ and *T*_sk_ were recorded online using a commercially available thermocouple metre (TC‐2000, Sable Systems International, Las Vegas, NV, USA) connected to a data acquisition system (PowerLab 26T, ADInstruments, Dunedin, New Zealand). Foot *T*_sk_ was collected via wireless temperature loggers (DS1922L iButton Thermochron, Measurement Systems Ltd, Newbury, UK). Following the protocol, foot temperature was exported from the wireless temperature loggers in 30‐s bins using a specialist logging software (iButtons, Measurement Systems Ltd). Data were then imported and analysed in Microsoft Excel software, reported as 2‐min averages. In addition, mean skin leg temperature (${\bar{T}}_{\mathrm{Leg}}$) was calculated as an unweighted average of quadriceps, hamstrings, calf and foot *T*_sk_. Similarly, mean skin upper‐leg temperature (${\bar{T}}_{\mathrm{Upper}-\mathrm{Leg}}$) was calculated as the unweighted average of quadriceps and hamstrings *T*_sk_, and mean skin lower‐leg temperature (${\bar{T}}_{\mathrm{Lower}-\mathrm{Leg}}$) was calculated as the unweighted average of calf and foot *T*_sk_.

### 2.5 Haemodynamic measurements

Heart rate was continuously measured using a three‐lead echocardiogram. Also, arterial blood pressure and cardiac output were measured non‐invasively – at the same time points as arterial blood flow measurements – using infrared photoplethysmography (Finometer, Finapres Medical Systems, Amsterdam, Netherlands), through a cuff on the middle finger of the right hand. Cardiac output was calculated as heart rate × stroke volume, where stroke volume was estimated using the ModelFlow method, which incorporated corrections for age, height and weight (Beatscope, Finapres Medical Systems) (Wesseling et al., 1993). Blood flow was measured at set time points – recording two 12‐s Doppler images – throughout the protocols in the various arteries using a duplex Doppler ultrasound system (Vivid E95, GE Healthcare, Chalfont St Giles, Buckinghamshire, UK) with a 9‐MHz linear array transducer probe (GE Healthcare) at an insonation angle of ≤60°, with sample volume positioned in the centre of the artery. The water‐perfusion heated trouser had custom‐made openings which allowed the probe to be placed on the skin with minimal heat loss. Before commencing baseline blood flow measures, arterial sites for the CFA, SFA and PFA in both legs were located and marked to ensure blood flow measurements were consistently made at the same site. SFA and PFA blood flow measurements were acquired at a distance of ≥2 cm from the femoral bifurcation to prevent turbulent flow disruption to the measurements, and thus improve validity of measures. Blood flow (mL min^−1^) was calculated using the following equation: $\mathrm{BF}\;=V_{\mathrm{mean}}\;\times \pi \;\times {\left(\frac{D_{\mathrm{mean}}}{2}\right)}^{2}\;\times \;60$, where *V*_mean_ is the average centreline blood velocity (cm s^−1^), and *D*_mean_ (cm) is the average internal diameter calculated using: $D_{\mathrm{mean}}=\frac{1}{3}\;\left(D_{\mathrm{systole}}\right)+\;\frac{2}{3}\left(D_{\mathrm{diastole}}\right)$ (Rådegran, 1997). It was not possible to directly measure PFA blood flow during the exercise protocol; therefore, during exercise, PFA blood flow was estimated using the following formula: PFAbloodflow=(CFA−SFA)bloodflow.

Shear rate (SR) was calculated using: $\mathrm{SR}\;=\frac{4\;\times \;V_{\mathrm{mean}}}{D_{\mathrm{mean}}}\;,$ where *V*_mean_ is the mean blood velocity. Additionally, vascular conductance (VC) was calculated using: VC=BF/MAP, where VC is in mL min^−1^ mmHg^−1^, BF is blood flow (mL min^−1^) and MAP is mean arterial pressure (mmHg). Blood flow was analysed offline using commercially available software (EchoPAC, GE Medical, Horton, Norway). Blood velocity was averaged over two 12‐s Doppler images, and average diameter was determined from four 2D B‐mode images. Furthermore, central haemodynamic and temperature data were collected at 1000 Hz using a commercially available data acquisition system (PowerLab 26T, ADInstruments) and exported in 30‐s bins using a commercially available data acquisition software (LabChart 7, ADInstruments). Following exportation, data were imported and analysed in Microsoft Excel. Data are reported as 2‐min averages.

### 2.6 Tissue oxygen saturation measures

Direct and continuous measurements of regional tissue haemoglobin oxygen saturation were obtained in the experimental and control legs using two near‐infrared spectroscopy units with four optodes each (NIRS; INVOS 5100C Cerebral Oximeter; Somanetics Corp, Troy, MI, USA). The optodes were placed on the skin surrounding the quadriceps, hamstrings, calves and feet of both legs and taped to reduce interference from external light sources.

### 2.7 Statistical analysis

Statistical analysis was conducted using R Studio (version 2022.07.1+554, R Core Team (2022)). A two‐way ANOVA was conducted to discover any differences in demographic and anthropometric data between the elderly and young cohorts, as well as identifying any potential anthropometric differences between legs. Moreover, three‐way repeated measures ANOVA was performed to investigate differences in haemodynamics, flow profiles, tissue oxygenation saturation and temperature between age cohorts and between and within the experimental and control legs over time during the passive heating protocol and between workloads during knee‐extensor exercise protocols, respectively. The repeated measures ANOVA was conducted following the confirmation of the data's normality via the Shapiro–Wilk test and Mauchly's test of sphericity. In addition, two‐way repeated measures ANOVA was conducted to investigate differences between age cohorts and over time in systemic variables – that is, heart rate, cardiac output, mean arterial pressure and core temperature – during the passive heating protocol. Following the two/three‐way repeated measures ANOVA, once a significant effect was found, Tukey's *post hoc* test was performed to locate the specific time points at which those changes occurred. Results are expressed as means ± SD. Significance is set at *P* < 0.05. Moreover, linear, exponential and polynomial regression curve fit tests were performed using GraphPad Prism (version 8, GraphPad Software, La Jolla, CA, USA) to assess the relationships among various key data. Subsequently, Akaike's information criterion was used to evaluate which model provides the most appropriate fit. Where an exponential curve fit was appropriate, the equation $y=y_{0}\;\times e^{kx}$ was used, where *y*_0_ is the *y* value (parameter investigated) when *x* (time) is zero and *k* is the rate constant. Significance of this fit is reported through 95% confidence intervals on the estimated value of *k* with the null value being k=0.

## 3 RESULTS

### 3.1 Demographic and anthropometric characteristics

Demographic and anthropometric data for the participants are reported in Table 1. Other than age (*P* < 0.0001), no differences were observed in height, mass, leg volume and lean mass between the two cohorts. Moreover, no anthropometric differences were found between the right and left legs in either group.

### 3.2 Effects of passive single‐leg heating on thermal, haemodynamic and tissue oxygen saturation responses in healthy aged and young participants

#### 3.2.1 Regional and core temperatures

**FIGURE 2:**
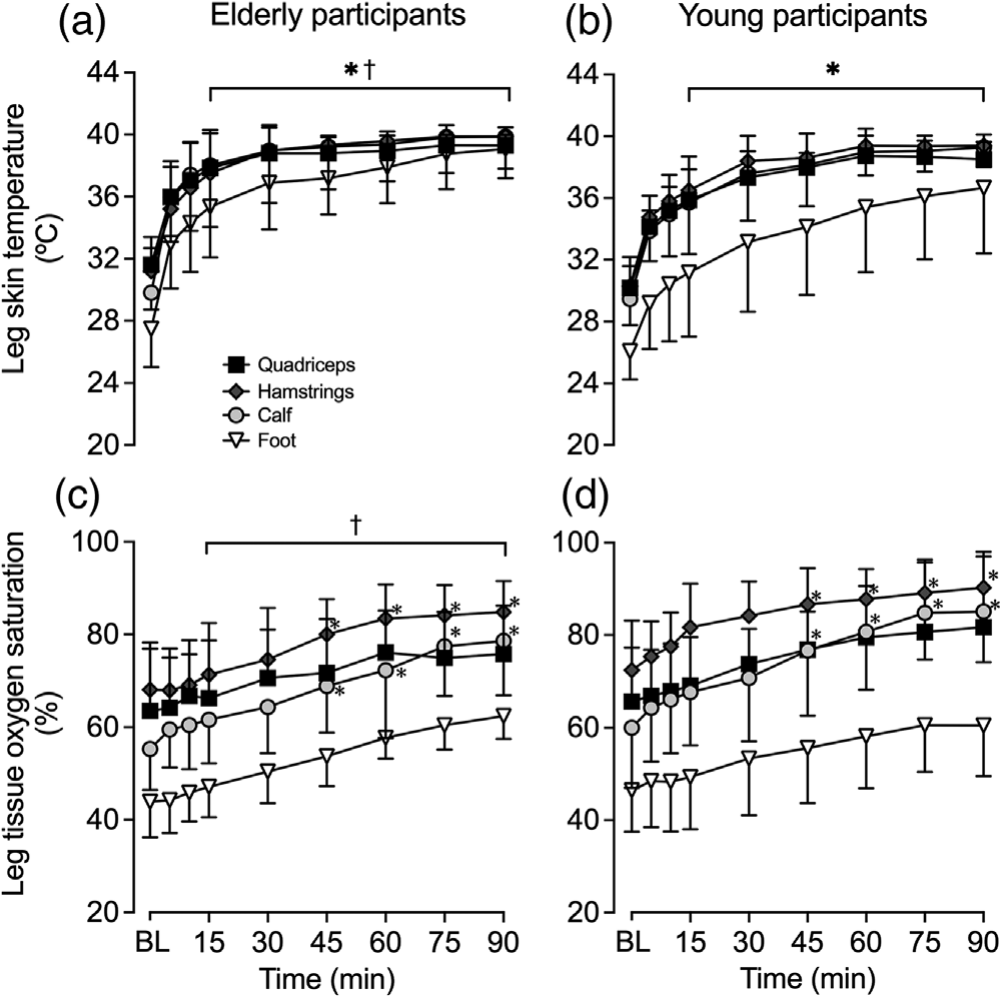
Heated (experimental) leg temperatures (a, b) and regional tissue oxygen saturation (c, d) during whole‐leg hyperthermia, in elderly (a, c) and young (b, d) participants. Data presented as means ± SD (elderly: *n* = 9; young: *n* = 10). BL signifies baseline measurements. *Different from baseline, *P* < 0.05. ^†^Different from control, young participants, *P* < 0.05.

Experimental leg *T*_sk_ values during passive heating—measured at the quadriceps, hamstrings, calf and foot—are illustrated in Figure 2, whilst *T*_c_ and ${\bar{T}}_{\mathrm{Leg}}$ for both the experimental and control legs are reported in Table 3. As per experimental design, leg *T*_sk_ progressively increased at all measured sites in the heated leg following 15 min of heating (all *P* < 0.0001), whereas ${\bar{T}}_{\mathrm{Leg}}$ for the control leg remained unchanged (*P* = 0.9989) (Table 3). Following 90 min of passive heating, both cohorts showed similar increases with ${\bar{T}}_{\mathrm{Leg}}$ for the experimental leg averaging 9.6 ± 1.3°C (*P* < 0.0001) in the aged cohort and 9.4 ± 1.2°C (*P* < 0.0001) in the young cohort, respectively (Figure 2). However, *T*_c_ remained unchanged throughout the entirety of the heating protocol (*P* = 0.9359) and was not different between cohorts (*P* = 0.7400) (Table 3).

**TABLE 2:** Influence of whole‐leg heating and subsequent one‐legged knee extensor exercise on body and skin temperatures and central haemodynamics.

		Passive leg heating					Control	
		Rest			One‐legged knee extensor exercise		One‐legged knee extensor exercise	
		Time (min)			Workload (W)		Workload (W)	
Intervention	Baseline	30	60	90	6	12	6	12
*T*_c_ (°C)								
Elderly	36.9 ± 0.4	36.9 ± 0.4	37.0 ± 0.4	37.2 ± 0.4	37.3 ± 0.4^†,‡^	37.3 ± 0.4^†,‡^	37.4 ± 0.4^†^	37.4 ± 0.4^†^
Young	37.1 ± 0.3	36.9 ± 0.2	36.9 ± 0.2	37.0 ± 0.2	37.1 ± 0.2	37.1 ± 0.2	37.2 ± 0.1	37.2 ± 0.1
${\bar{T}}_{\mathrm{Leg}}$ (°C)								
Experimental leg								
Elderly	30.0 ± 1.4^†^	38.3 ± 2.1^*,†,‡^	38.9 ± 1.4^*,†,‡^	39.6 ± 1.2^*,†,‡^	38.2 ± 1.3^‡^	37.4 ± 1.0^‡^	33.9 ± 0.9^‡^	33.3 ± 0.9^‡^
Young	29.1 ± 1.3	36.7 ± 1.5^*^	38.2 ± 1.3^*^	38.5 ± 1.1^*^	37.9 ± 1.4	37.7 ± 1.6	33.7 ± 0.7	33.3 ± 0.7
Control leg								
Elderly	29.9 ± 1.5	29.6 ± 1.5	29.3 ± 1.5	29.1 ± 1.5	28.6 ± 1.2	28.6 ± 1.3	28.7 ± 1.3	29.1 ± 1.7
Young	28.7 ± 0.9	28.2 ± 0.8	28.1 ± 0.8	28.2 ± 0.9	28.1 ± 1.2	28.0 ± 1.3	28.2 ± 1.2	28.4 ± 1.2
MAP (mmHg)								
Elderly	93 ± 12	91 ± 14	91 ± 8	87 ± 13	115 ± 19^*^	110 ± 20^*^	122 ± 22^*^	121 ± 20^*^
Young	98 ± 22	94 ± 12	95 ± 13	92 ± 13	111 ± 24^*^	112 ± 26^*^	118 ± 25^*^	119 ± 24^*^
$\dot{Q}$ (L min^−1^)								
Elderly	4.5 ± 1.1^†^	4.0 ± 1.2^†^	4.7 ± 2.1^†^	4.7 ± 1.9^†^	6.2 ± 1.6^*,†^	8.0 ± 0.8^*,†^	5.3 ± 1.2^*,†^	6.4 ± 1.7^*,†^
Young	5.0 ± 1.0	5.7 ± 0.8	5.5 ± 0.7	5.8 ± 0.7	7.9 ± 1.9^*^	8.9 ± 2.0^*^	7.4 ± 1.6^*^	8.1 ± 1.8^*^
HR (beats min^−1^)								
Elderly	57 ± 10^†^	58 ± 12^†^	56 ± 10^†^	61 ± 12^†^	81 ± 24^*,†^	79 ± 19^*,†^	80 ± 26^*,†^	80 ± 22^*,†^
Young	66 ± 9	62 ± 12	64 ± 9	69 ± 9	92 ± 22^*^	100 ± 24^*^	89 ± 26^*^	96 ± 32^*^

Values are means ± SD for nine elderly participants and 10 young participants. Experimental leg refers to right, heated leg, whilst the control leg refers to the left, contralateral leg. Values for rest, represent the responses during 90 min of passive whole‐leg heating. Leg skin temperature is presented as an unweighted mean average collected at four different sites: quadriceps, hamstrings, calf and foot. ^*^Different from baseline, *P* < 0.05. ^†^Different from respective young, control group at the same time point, *P* < 0.05. ^‡^Different from respective contralateral, control leg, *P* < 0.05. Abbreviations: HR, heart rate; MAP, mean arterial pressure; ${\bar{T}}_{\mathrm{Leg}}$, mean leg skin temperature; $\dot{Q}$, cardiac output.

#### 3.2.2 Leg blood flow, tissue oxygen saturation and systemic haemodynamics

**FIGURE 3:**
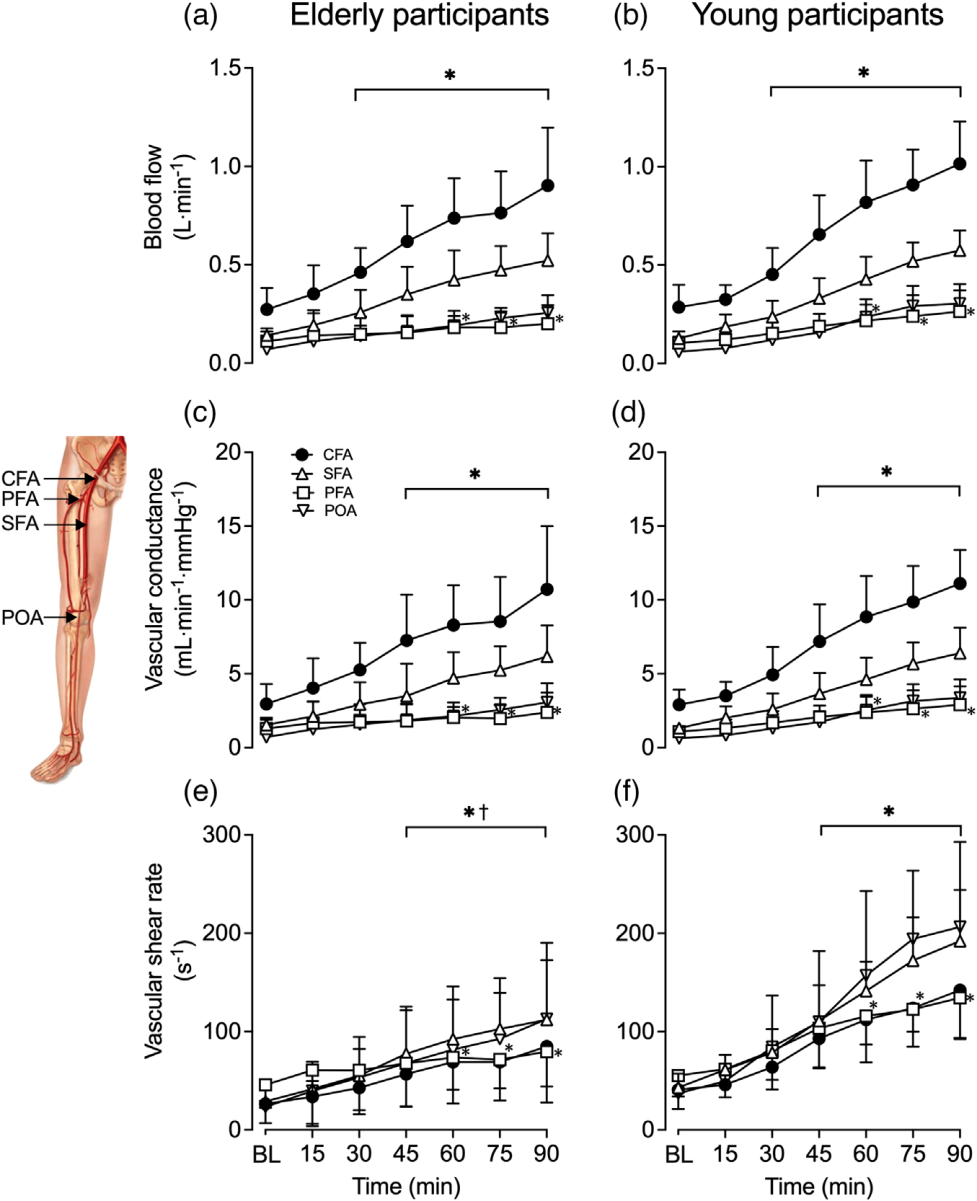
Blood flow (a, b), vascular conductance (c, d) and shear rate (e, f) during whole‐leg hyperthermia in the common (CFA), superficial (SFA) and profunda (PFA) femoral arteries and the popliteal (POA) of the heated (experimental) leg, in elderly (a, c, e) and young (b, d, f) participants. Data presented as means ± SD (elderly: *n* = 9; young: *n* = 10). BL signifies baseline measurements. *Different from baseline, *P* < 0.05. ^†^Different from control, young participants, *P* < 0.05.

**FIGURE 4:**
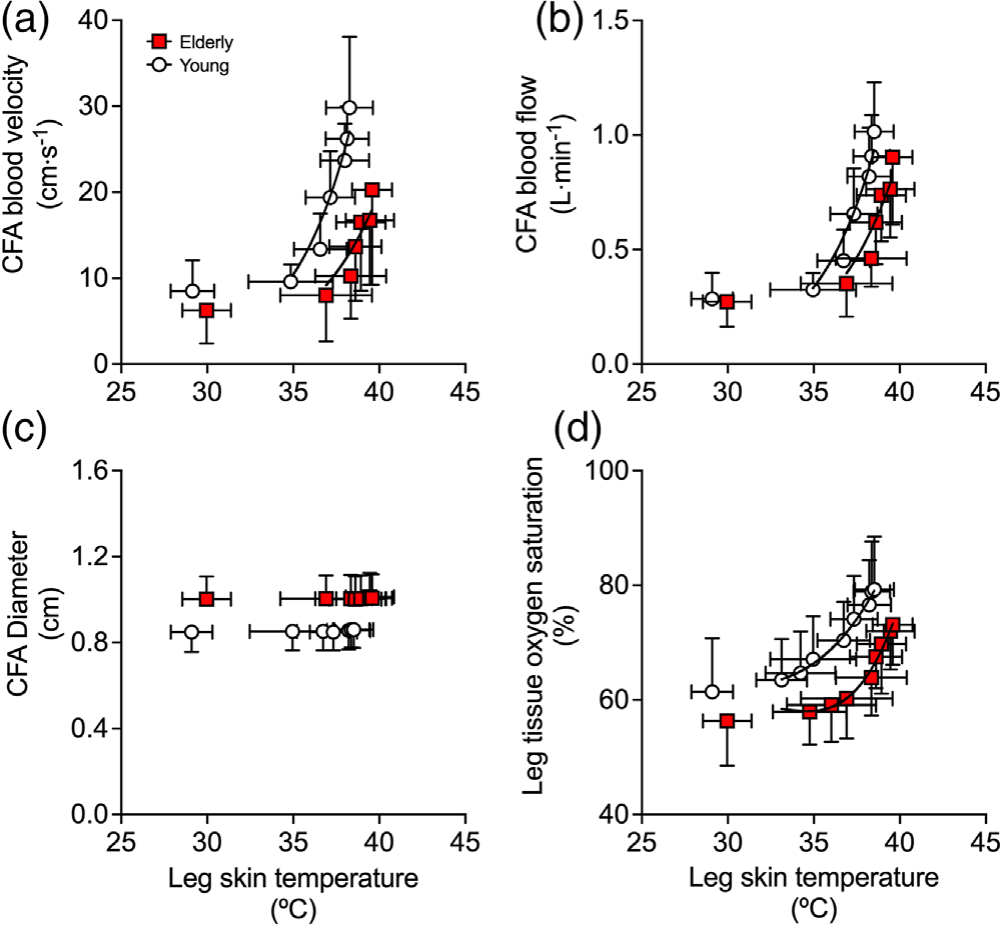
Relationship between the mean leg skin temperature common femoral artery (CFA) blood velocity (a), blood flow (b), diameter (c) and mean leg tissue oxygen saturation (d) during whole‐leg hyperthermia in elderly and young participants. Data presented as means ± SD (elderly: *n* = 9; young: *n* = 10). Vertical error bars signify local blood flow SD, while horizontal error bars signify local temperature SD. Lines represent the exponential fit of the data.

**FIGURE 5:**
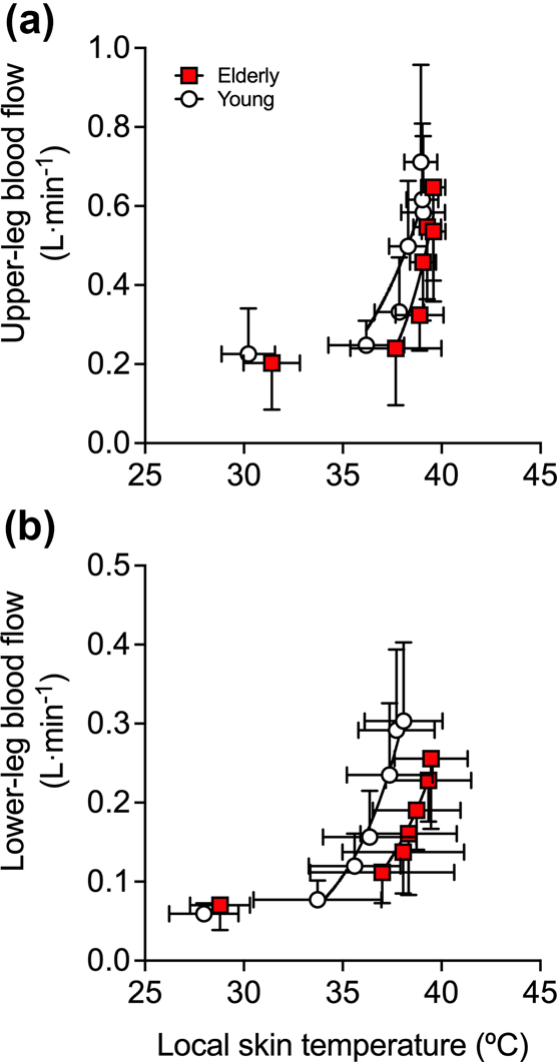
Relationship between the local skin temperature and local blood flow during whole‐leg hyperthermia in elderly and young participants. Data presented as means ± SD (elderly: *n* = 9; young: *n* = 10). The graphs illustrate the relationship between upper‐leg blood flow and upper‐leg skin temperature (a), and lower‐leg blood flow and lower‐leg skin temperature (b). Vertical error bars signify local blood flow SD, while horizontal error bars signify local temperature SD. Lines represent the exponential fit of the data.

Complete haemodynamic responses during 90 min of passive whole‐leg heating for the CFA, SFA, PFA and POA of the experimental leg are reported in Figure 3, whilst whole‐leg blood flow for the control leg is reported in Table 2. In the heated leg, blood flow in the CFA, SFA and POA increased progressively up to values ≥3.5‐fold above baseline (∆ = 0.68 ± 0.21, 0.42 ± 0.11 and 0.22 ± 0.10 L min^−1^, respectively), whilst PFA blood flow increased ∼2‐fold (∆ = 0.11 ± 0.09 L min^−1^) following 90 min (all *P* < 0.0001). Elevations in whole‐leg arterial blood velocity, blood flow and tissue oxygen saturation were exponentially related to increases in leg skin temperature—for both the aged participants (CFA blood velocity: *R* ^2^ = 0.75, *k* = 0.25 (0.04, 0.71); CFA blood flow: *R* ^2^ = 0.77, *k* = 0.27 (0.05, 0.70); tissue oxygen saturation: *R* ^2^ = 0.74, *k* = 0.03 (0.01, 0.05)) and young participants (CFA blood velocity: *R* ^2^ = 0.85, *k* = 0.29 (0.10, 0.64); CFA blood flow: *R* ^2^ = 0.86, *k* = 0.29 (0.10, 0.62); tissue oxygen saturation *R* ^2^ = 0.88, *k* = 0.03 (0.02, 0.04)) (Figure 4). No relationships were observed between leg tissue skin temperature and diameter, which remained unchanged in all measured arteries (all *P* ≥ 0.948) (Figure 4). Moreover, segmental blood flow (i.e. upper‐leg and lower‐leg) was exponentially related to increases in local skin temperature (upper leg: *R* ^2^ = 0.68, *k* = 0.46 (0.03, 1.33); lower leg: *R* ^2^ = 0.84, *k* = 0.27 (0.07, 0.56)) and young participants (upper leg: *R* ^2^ = 0.74, *k* = 0.27 (0.05, 0.85); lower leg: *R* ^2^ = 0.94, *k* = 0.34 (0.20, 0.53)) (Figure 5). Arterial blood flow increased in parallel to blood velocity – ≥3.5‐fold in CFA, SFA and POA and ∼2‐fold in PFA (all *P* < 0.0001). Despite no changes in arterial diameter over time, differences in absolute diameter were observed between the two age cohorts for all arteries, with the elderly cohort showing a 16 ± 6% larger diameter (*P* < 0.0001) average obtained by comparing diameters across all arteries at baseline for both legs (Table 2). Correspondingly, the elderly cohort demonstrated a 25 ± 12% lower blood velocity at baseline conditions averaged across all arteries, with the difference between cohorts further increasing to 51 ± 6% following 90 min of heating (*P* < 0.0001) (Table 2). Similar responses were observed in vascular conductance and SR in all four arteries of the heated leg (Figure 3). Vascular conductance and SR increased ≥3.4‐fold in the CFA, SFA and POA and ≥2.1‐fold in the PFA (all *P* < 0.0001). No changes in control leg blood flow and vascular conductance were observed in all arteries (*P ≥* 0.9999), nor were there any differences in arterial blood flow (*P* ≥ 0.0720) or vascular conductance (*P* ≥ 0.5030) between the cohorts. However, the elderly cohort showed a 51 ± 7% lower SR in all arteries during baseline conditions, which was further exacerbated to 73 ± 7% following 90 min of heating (all *P* < 0.0001), associated with a 16 ± 6% larger diameter.

**TABLE 3:** Influence of whole‐leg heating and subsequent one‐legged knee extensor exercise on arterial blood flow and tissue oxygen saturation.

		Passive leg heating					Control	
		Rest			One‐legged knee extensor exercise		One‐legged knee extensor exercise	
		Time (min)			Workload (W)		Workload (W)	
Intervention	Baseline	30	60	90	6	12	6	12
CFA blood flow (L min^−1^)								
Experimental leg								
Elderly	0.27 ± 0.10	0.46 ± 0.12^*,‡^	0.74 ± 0.20^*,‡^	0.90 ± 0.30^*,‡^	2.93 ± 0.48^*,‡^	3.31 ± 0.49^*,‡^	—	—
Young	0.29 ± 0.11	0.45 ± 0.13^*^	0.82 ± 0.21^*^	1.02 ± 0.22^*^	2.42 ± 0.83^*,‡^	2.96 ± 0.70^*,‡^	—	—
Control leg								
Elderly	0.29 ± 0.08	0.26 ± 0.07	0.24 ± 0.08	0.24 ± 0.09	—	—	2.15 ± 0.52^*,‡^	2.34 ± 0.39^*,‡^
Young	0.29 ± 0.10	0.27 ± 0.09	0.26 ± 0.07	0.27 ± 0.08	—	—	1.87 ± 0.49^*^	2.24 ± 0.43^*^
CFA blood velocity (cm s^−1^)								
Experimental leg								
Elderly	6.3 ± 3.9^†^	10.3 ± 5.0^†,‡^	16.5 ± 8.0^*,†,‡^	20.3 ± 11.0^*,†,‡^	63.8 ± 19.9^*,†,‡^	70.1 ± 8.9^*,†,‡^	—	—
Young	8.5 ± 3.6	13.4 ± 4.2	23.7 ± 4.3	29.8 ± 8.3	67.4 ± 26.3^*,‡^	83.3 ± 23.3^*,‡^	—	—
Control leg								
Elderly	6.3 ± 2.2^†^	5.7 ± 1.8^†^	5.4 ± 2.3^†^	5.6 ± 3.3^†^	—	—	48.5 ± 16.4^*,†^	51.5 ± 9.2^*,†^
Young	8.3 ± 2.9	7.7 ± 2.3	7.7 ± 2.1	7.8 ± 1.7	—	—	52.3 ± 17.1^*^	62.1 ± 13.4^*^
CFA diameter (cm)								
Experimental leg								
Elderly	1.00 ± 0.10^†^	1.00 ± 0.11^†^	1.00 ± 0.11^†^	1.01 ± 0.11^†^	1.01 ± 0.10^†^	1.00 ± 0.04^†^	—	—
Young	0.85 ± 0.09	0.85 ± 0.09	0.85 ± 0.09	0.86 ± 0.09	0.88 ± 0.08	0.88 ± 0.08	—	—
Control leg								
Elderly	1.00 ± 0.10^†^	0.99 ± 0.10^†^	0.99 ± 0.10^†^	0.99 ± 0.10^†^	—	—	0.99 ± 0.10^†^	0.98 ± 0.05^†^
Young	0.86 ± 0.10	0.86 ± 0.09	0.86 ± 0.09	0.86 ± 0.10	—	—	0.88 ± 0.08	0.88 ± 0.08
Tissue oxygen saturation (%)								
Experimental leg								
Elderly	56 ± 8^†^	64 ± 6^*,†,‡^	70 ± 9^*,†,‡^	73 ± 7^*,†,‡^	69 ± 6^†,‡^	68 ± 6^†,‡^	71 ± 9^†,‡^	71 ± 9^†,‡^
Young	61 ± 9	70 ± 7^*^	77 ± 8^*^	79 ± 9^*^	76 ± 6	76 ± 6	79 ± 8	78 ± 8
Control leg								
Elderly	57 ± 8^†^	57 ± 7^†^	55 ± 10^†^	59 ± 8^†^	56 ± 9^†^	56 ± 8^†^	54 ± 6^†^	50 ± 13^†^
Young	62 ± 8	64 ± 6	65 ± 5	67 ± 6	64 ± 9	64 ± 8	65 ± 7	65 ± 7

Values are means ± SD for nine elderly participants and 10 young participants. Experimental leg refers to right, heated leg, whilst the control leg refers to the left, contralateral leg. Tissue oxygen saturation reflects changes in regional tissue haemoglobin oxygen saturation. Values for rest, represent the responses during 90 min of passive whole‐leg heating. ^*^Different from baseline, *P* < 0.05. ^†^Different from respective young, control group at the same time point, *P* < 0.05. ^‡^Different from respective contralateral, control leg, *P* < 0.05. Abbreviation: CFA, common femoral artery.

Tissue haemoglobin oxygen saturation increased gradually at all four sites (quadriceps, hamstrings, calf and foot) of the heated leg, with mean leg tissue oxygen saturation increasing 17 ± 6% units (*P* < 0.0001) in the aged cohort and 18 ± 6% units (*P* < 0.0001) in the young cohort following 90 min of heating (Figure 2 and Table 2). No differences in tissue oxygen saturation were observed in the control leg (*P* = 0.9531). However, differences between the age cohorts were observed, with the elderly cohort exhibiting a lower mean leg tissue oxygen saturation in the heated leg (73 ± 7 vs. 79 ± 9%; *P* < 0.0001) and control leg (59 ± 8 vs. 67 ± 6%; *P* < 0.0001). At the systemic haemodynamic level, no changes were observed for heart rate (*P* = 0.6465), cardiac output (*P* = 0.9390) and mean arterial pressure (*P* = 0.8920) during 90 min of heating (Table 3). However, heart rate (*P* = 0.0006) and cardiac output (*P* < 0.0001) were lower in the aged cohort in comparison to their younger counterparts (Table 3).

### 3.3 Effects of incremental low‐intensity knee extensor exercise and single‐leg heating on thermal, hemodynamic and tissue oxygen saturation responses in healthy aged and young participants

#### 3.3.1 Regional and core temperatures

**FIGURE 6:**
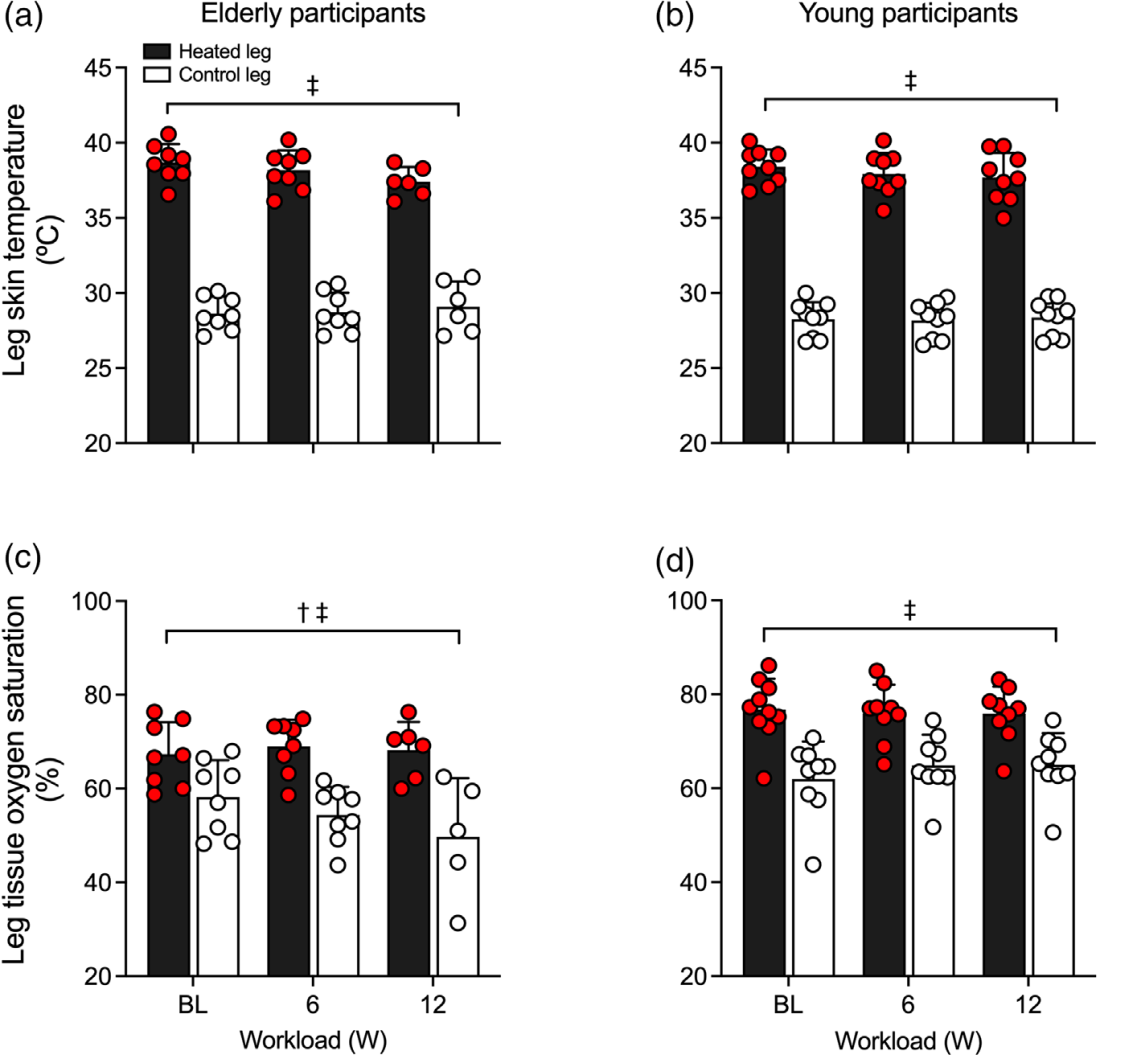
Heated (experimental) leg skin temperature (a, b) and tissue oxygen saturation (c, d) during one‐legged knee extensor exercise with heating, in elderly (a, c) and young (b, d) participants. Data presented as means ± SD (elderly: *n* = 9; young: *n* = 9). Circles symbolise individual data points. Baseline (BL) measurements signify the measurements taken immediately prior to the start of exercise, following 90 min of whole‐leg heating. Leg skin temperature and tissue oxygen saturation are presented as an unweighted mean average collected at four different sites: quadriceps, hamstrings, calf and foot. *Different from baseline, *P* < 0.05. ^†^Different from control, young participants, *P* < 0.05.

As per the experimental design, heated leg *T*_sk_ was substantially higher at all measured sites in comparison to the control leg (*P* < 0.0001) (Figure 6 and Table 3). When comparing the two legs during their respective exercise protocols, ${\bar{T}}_{\mathrm{Leg}}\;$was 9.3 ± 1.5°C higher in the heated leg (*P* < 0.0001). Additionally, no differences were observed in ${\bar{T}}_{\mathrm{Leg}}$ between workloads (*P* = 0.345) or age cohorts (*P* = 0.164) (Figure 6 and Table 3). Conversely, small but significant differences in *T*_c_ were observed between leg exercise protocols and age cohorts: *T*_c_ was 0.1 ± 0.1°C lower during the heated leg exercise protocol in comparison to the control leg exercise protocol (*P* = 0.0441), and 0.2 ± 0.1°C higher in the aged cohort (*P* = 0.0143) (Table 3).

#### 3.3.2 Leg blood flow, tissue oxygen saturation and systemic haemodynamics

**FIGURE 7:**
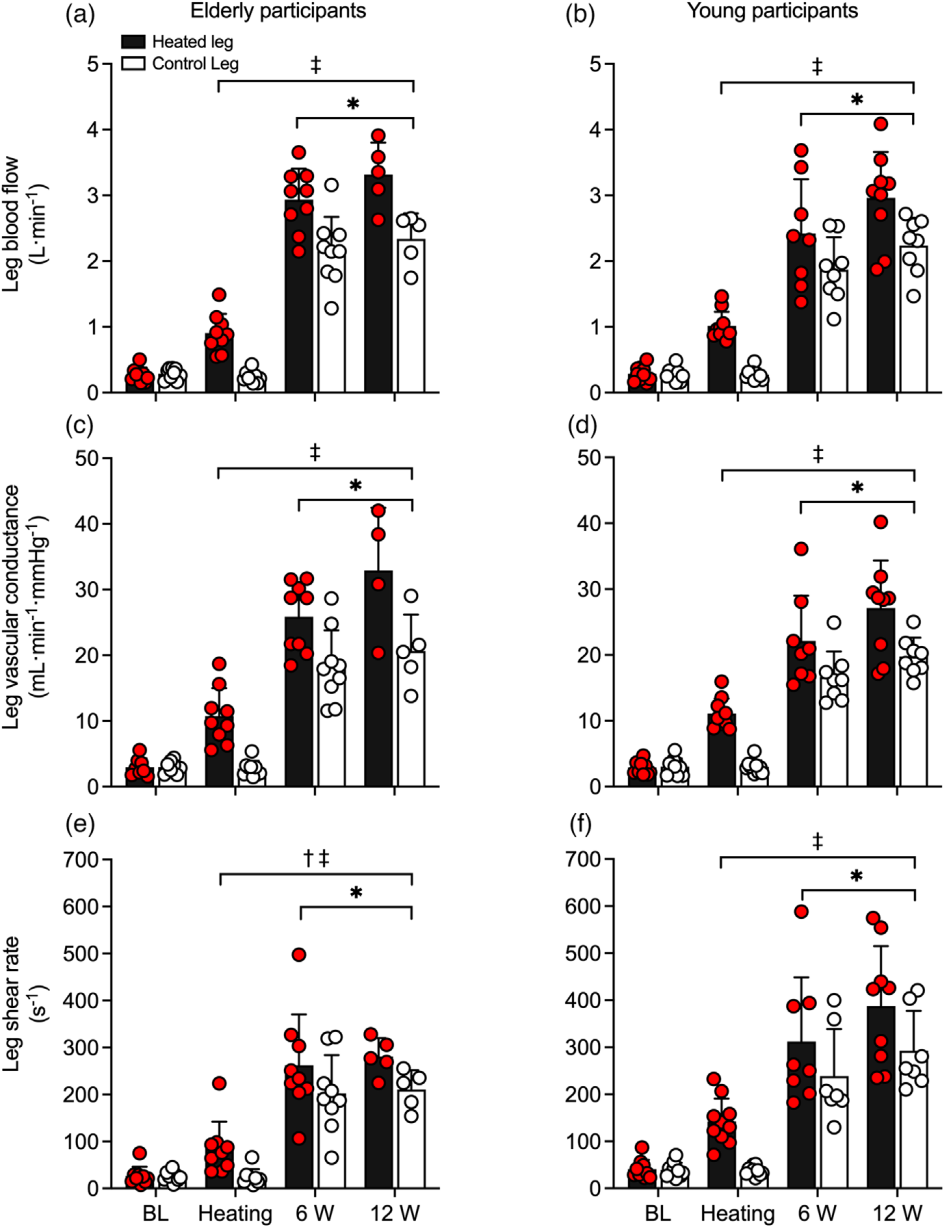
Blood flow (a, b), vascular conductance (c, d), and shear rate (e, f) during one‐legged knee extensor exercise with and without heating, in the common femoral artery of the heated (experimental) leg and control (contralateral) leg, in elderly (a, c, e) and young (b, d, f) participants. Data presented as means ± SD (elderly: *n* = 9; young: *n* = 9). Circles symbolise individual data points. *Different from baseline (BL) prior to the commencement of exercise (measurements taken following 90 min of whole‐leg heating), *P* < 0.05. ^†^Different from respective young, control group at the same time point, *P* < 0.05. ^‡^Different from respective contralateral, control leg, *P* < 0.05.

**FIGURE 8:**
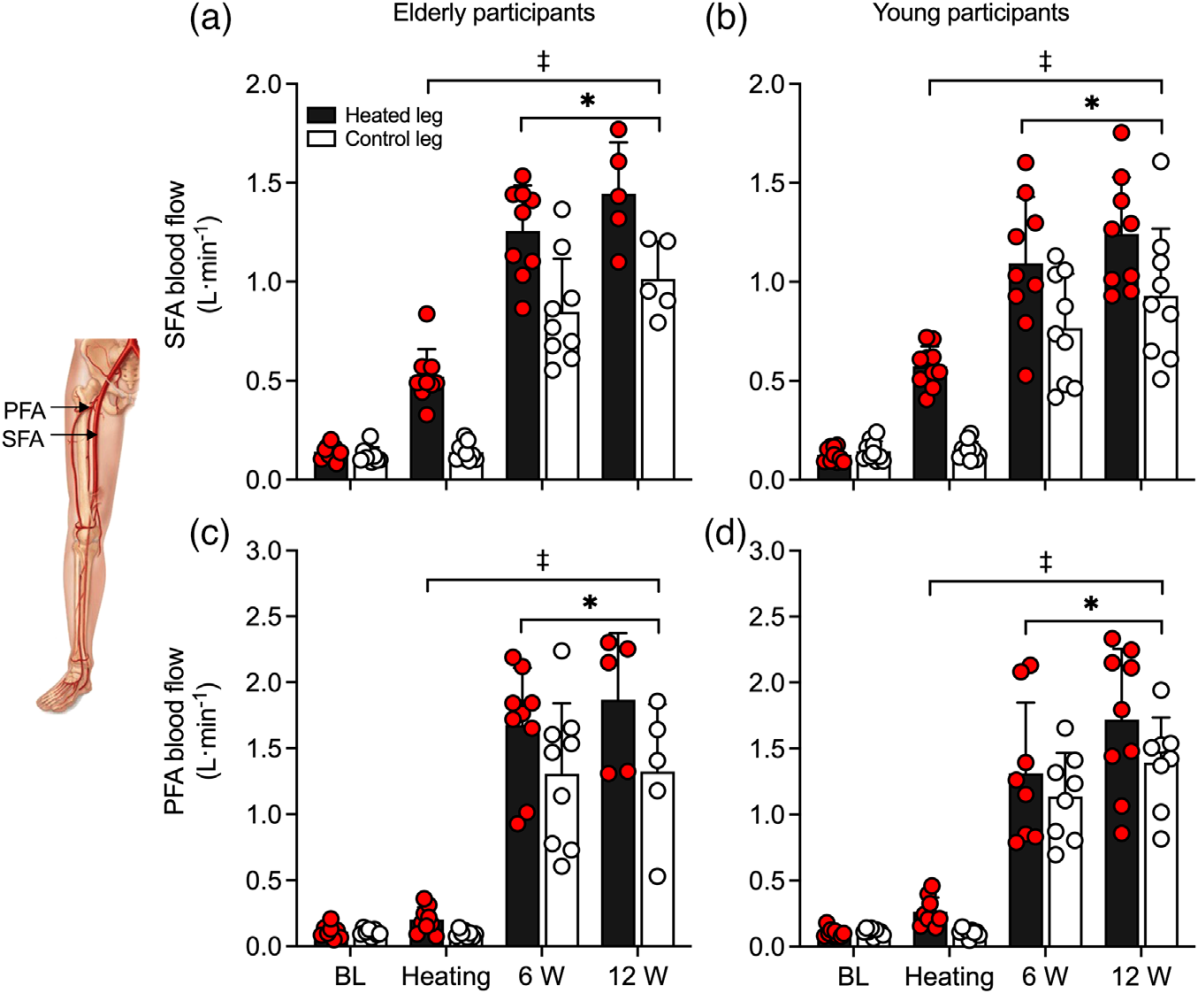
Blood flow during one‐legged knee extensor exercise with and without heating, superficial (SFA) (a, b) and profunda (PFA) (c, d) femoral arteries of the heated (experimental) leg and control (contralateral) leg, in elderly (a, c) and young (b, d) participants. Data presented as means ± SD (elderly: *n* = 9; young: *n* = 9). Circles symbolise individual data points. Note: during exercise, PFA blood flow was calculated indirectly as the difference between CFA and SFA blood flow. *Different from baseline (BL) prior to the commencement of exercise (measurements taken following 90 min of whole‐leg heating), *P* < 0.05. ^†^Different from respective young, control group at the same time point, *P* < 0.05. ^‡^Different from respective contralateral, control leg, *P* < 0.05.

Whole‐leg (CFA) haemodynamics during exercise are reported in Figure 7 and Table 2, whilst SFA and PFA blood flows are reported in Figure 8. In absolute terms, CFA blood flow in the heated leg was 0.65 ± 0.56 and 1.04 ± 0.83 L min^−1^ higher at 6 and 12 W, respectively, in comparison to the control exercise condition (Figure 7). Similar responses were observed in the SFA and PFA, with heated exercise inducing 0.36 ± 0.18 and 0.36 ± 0.30 L min^−1^ higher blood flows than the control exercise for the SFA at 6 and 12 W, respectively, and 0.28 ± 0.49 and 0.36 ± 0.53 L min^−1^ for the PFA at 6 and 12 W, respectively (all *P* < 0.0001). However, no differences were observed between age cohorts (*P* ≥ 0.1131). As reported earlier, arterial diameter did not change during the protocol (*P* ≥ 0.700), although the average diameter was larger in the elderly cohort (*P* < 0.0001). Whole‐leg vascular conductance was 7 ± 5 and 8 ± 7 mL min^−1^ mmHg^−1^ higher in the heated leg compared to the control leg during 6 and 12 W of exercise, respectively (Figure 7). No differences were observed for vascular conductance between the age cohorts (*P* = 0.1540). Likewise, CFA SR was 61 ± 65 and 73 ± 60 s^−1^ higher in the heated leg during 6 and 12 W of exercise, respectively (Figure 7). Lastly, differences were observed between legs (*P* < 0.0001) and between cohorts (*P* = 0.0011), with the elderly cohort reporting a reduced SR across both exercise workloads and legs (Figure 7).

No differences were observed in mean leg tissue oxygen saturation during exercise, across both workloads (*P* = 0.9880). However, differences were observed between legs and between age cohorts, with the heated leg showing a higher mean leg tissue oxygen saturation across both workloads (73 ± 7 vs. 51 ± 8%, respectively, *P* < 0.0001) and the elderly cohort reporting a lower mean leg tissue oxygen saturation during heated‐leg exercise (76 ± 6 vs. 69 ± 6%, respectively, *P* < 0.0001) (Figure 6 and Table 2). At the systemic haemodynamic level, no differences in arterial pressure or cardiac output were observed between the heated‐ and control‐leg exercise protocols (all *P* ≥ 0.2160) (Table 3). However, differences were observed between age cohorts for heart rate (*P* = 0.0122) and cardiac output (*P* < 0.0001), with the elderly cohort reporting lower values during the exercise protocols (Table 3).

## 4 DISCUSSION

The present study sought to characterise the haemodynamic profiles of the major leg arteries in response to single‐leg hyperthermia, single‐leg knee‐extensor exercise, and their combination in healthy, active elderly and young participants and in doing so, gain insight into the impact of local temperature and age on the control of limb perfusion. In line with our primary hypothesis, blood flow was tightly coupled to the rise in local temperature and metabolic demand, with the combination of local limb hyperthermia and exercise producing a higher level of hyperaemia than exercise alone. However, contrary to our secondary hypothesis, no differences in the hyperaemic response to local limb hyperthermia, normal exercise or hyperthermic exercise were observed between the elderly and young cohort. Importantly, the equal hyperaemia happened despite the elderly group exhibiting a lower blood velocity during all three interventions. Collectively, the present findings suggest that age indeed induces structural and functional vascular adaptation—as shown by the age‐related differences in arterial diameter, blood velocity and shear stress – but it does not alter the magnitude of hyperaemia during local limb hyperthermia, small muscle mass exercise or combined limb hyperthermia and exercise.

### 4.1 Effect of hyperthermia on the hyperaemic response to low‐intensity knee‐extensor exercise in old and young humans

An important finding of this study is that local hyperthermia augmented macro‐ and microcirculatory blood flow similarly at rest and during single‐leg exercise, irrespective of age. Ninety minutes of passive single‐leg heating increased ${\bar{T}}_{\mathrm{Leg}}$ by 9.5°C, inducing an average 0.6–0.7 L min^−1^ elevation in whole‐leg blood flow in the elderly and young cohorts. The relationships between the rise in local temperature (>35°C skin temperature) and tissue perfusion for both the upper and the lower leg in both groups are consistent with previous findings from our laboratory during isolated leg heating in young participants (Figure 5) (Chiesa et al., 2016; Koch Esteves et al., 2021; Pearson et al., 2011). Similarly, the addition of single‐leg hyperthermia during knee‐extensor exercise in both cohorts produced a 0.7 L min^−1^ larger leg hyperaemia at 6 W and 1.0 L min^−1^ at 12 W, in comparison to exercise alone. Functional hyperaemia during control‐leg exercise was also independent of age, as whole‐leg blood flow was comparable between the elderly and young cohorts—2.2 versus 1.9 L min^−1^ and 2.3 versus 2.2 L min^−1^ at 6 and 12 W, respectively (Figure 7). The magnitude of the local hyperthermia‐induced hyperaemia was, therefore, remarkably similar at rest and during single‐leg knee‐extensor exercise in both groups.

An age‐old question is whether heat stress provides an additive stimulus for functional hyperaemia, with the premise that tissue temperature and perfusion are elevated compared to control conditions. Evidence to date is equivocal, however, with several past studies reporting no differences in leg blood flow during two‐legged cycling exercise (Savard et al., 1988; Trangmar et al., 2017) and single‐leg knee extensor exercise (Ferguson et al., 2006; Savard et al., 1988), whilst others reported an increased flow of 0.6–0.7 L min^−1^ between hyperthermic and control conditions (Chiesa et al., 2015; Pearson et al., 2011). Exercise duration, intensity and mass of active muscle all influence the balance between heat production and heat transfer, which ultimately affect local muscle and blood temperatures (González‐Alonso et al., 2015, 2000; Saltin & Hermansen, 1966; Saltin et al., 1968). Since local tissue perfusion is strongly related to local skin and muscle temperature (Koch Esteves et al., 2021), it is plausible that some of the aforementioned studies did not find an effect of environmental heat stress or external heating on functional hyperaemia because these interventions did not sufficiently increase deep tissue temperature above the control exercise condition (Ferguson et al., 2006; Savard et al., 1988; Trangmar et al., 2017). In this light, it is important to consider that whole‐leg exercise – for example, cycling – increases tissue and blood temperatures in the whole leg whereas knee‐extensor exercise increases temperature solely in the quadriceps muscle (González‐Alonso et al., 2000). Localised thigh heating, on the other hand, increases temperature and perfusion only in the upper‐leg segment (Koch Esteves et al., 2021). Thus, the larger differences in tissue temperature across the whole leg between the present heated and control exercise protocols likely rationalise the additive effect of leg hyperthermia on functional hyperaemia.

While the influence of combined local‐leg heating and exercise on whole‐leg hyperaemia was consistent in both groups, the precise distribution of the additional exertional hyperaemia remains uncertain. Assuming that lower‐leg perfusion—supplied by the POA—remained stable during knee‐extensor exercise, which following passive leg heating increased by ∼0.2 L min^−1^ (Figure 3), it is estimated that upper‐leg hyperaemia was 0.5–0.8 L min^−1^ higher during combined leg hyperthermia and exercise at the two workloads examined, in comparison to control exercise. Additionally, if the hyperthermia‐induced hyperaemia is equally distributed among the thigh tissues, we estimated that 0.2–0.4 L min^−1^ would be perfusing the anterior thigh tissues, with a significant portion added to the normal exercise hyperaemia perfusing the active quadriceps muscle, which would amount to a 9–18% elevation in exertional hyperaemia. Evidence in the literature supports this argument. On one hand, muscle perfusion and tissue oxygen saturation increase and leg arteriovenous oxygen difference declines in response to passive local hyperthermia (Chiesa et al., 2015; Heinonen et al., 2011; Keller et al., 2010; Pearson et al., 2011). Moreover, leg (quadriceps) ${\dot{V}}_{O_{2}}$ is maintained during knee‐extensor exercise under hyperthermic conditions in association with compensatory reductions in arteriovenous oxygen differences (Chiesa et al., 2015; Pearson et al., 2011). The higher tissue oxygen saturation seen in this study during leg heating and knee‐extensor exercise suggests that a higher muscle hyperaemia and corresponding increased oxygen delivery were met by a lower oxygen extraction from the circulation in both the elderly and young cohorts (Figure 5). Together, these findings reveal that hyperthermia induces increases in muscle perfusion during knee‐extensor exercise despite an unchanged metabolic demand. Furthermore, these observations lend support to the notion that the rise in local temperature is a putative stimulus for augmenting muscle tissue perfusion during low‐intensity small muscle mass exercise in both young and elderly individuals.

### 4.2 Effect of age on the thermal mechanisms enhancing skeletal muscle perfusion during low‐intensity knee‐extensor exercise

The literature surrounding the effect of age on functional hyperaemia in response to hyperthermia and exercise is riddled with inconsistencies, yet the prevailing view is that age attenuates functional hyperaemia. According to Darcy's law of flow – the hydraulic equivalent of Ohm's law – skeletal muscle perfusion is determined by vascular conductance and perfusion pressure gradient. Using mean arterial pressure as a surrogate, no differences in the perfusion pressure gradient were observed during single‐leg heating and between the elderly and young cohorts (Table 3). Hence, hyperperfusion during single‐leg heating was solely associated with increases in vascular conductance (Figure 3). A blunted vascular conductance is commonly reported in elderly individuals during hyperthermia and exercise and postulated as a primary mediator of the age‐associated attenuation in tissue perfusion (Kenney, 2001; Martin et al., 1995; Hearon Jr & Dinenno, 2016). Reduced bioavailability of endothelium‐derived vasoactive substances (i.e. nitric oxide) and compromised functional sympatholysis – that is, a reduced ability to counteract vasoconstriction and maintain adequate blood flow in the presence of elevated sympathetic nerve activity – have been implicated in this phenomenon (Hearon Jr & Dinenno, 2016). From a global haemodynamic perspective, an age‐related attenuation in hyperaemia during local lower‐leg hyperthermia (Romero et al., 2017) and knee‐extensor exercise (Donato et al., 2006; Lawrenson et al., 2003; Mortensen et al., 2012) has been associated with a lower vascular conductance in elderly sedentary and normally active subjects, which in some cases is largely the result of an elevated mean arterial pressure (Donato et al., 2006; Lawrenson et al., 2003; Mortensen et al., 2012). In contrast, the present elderly cohort demonstrated a comparable vascular conductance, mean arterial pressure and blood flow to their younger counterparts (Figure 7) in line with recent studies during local two‐leg hyperthermia (Engelland et al., 2020) and submaximal knee‐extensor exercise in endurance‐trained aged cohorts (Mortensen et al., 2012). The discrepancies among studies may be explained, at least in part, by regular participation in physical activity, which has been shown to preserve arterial responsiveness to vasodilator infusion and functional sympatholysis with age (Kruse et al., 2018; Mortensen et al., 2012; Piil et al., 2018). Alternatively, functional sympatholysis may not impose a limitation on the hyperaemic response in the present study because – unlike two‐leg heating (Engelland et al., 2020), whole‐body hyperthermia (Crandall et al., 1999; Cui et al., 2004) and two‐legged cycling (Katayama & Saito, 2019) – single lower‐leg heating and moderate‐intensity dynamic knee‐extensor exercise (30 W) consistently or transiently reduce muscle sympathetic nerve activity compared to baseline values (Katayama & Saito, 2019; Takahashi et al., 2011). Moreover, circulating noradrenaline levels remain low (∼1–2 nmol L^−1^) when core temperature is unchanged during heating and moderate intensity single‐leg knee‐extensor exercise (Chiesa et al., 2019; Pearson et al., 2011; Powers et al., 1982). It therefore appears that lifelong regular engagement in physical activity and the low vasoconstrictor activity during single‐leg hyperthermia and exercise contribute to the preservation of functional hyperaemia during local limb heating and small muscle mass exercise.

Increases in leg vascular conductance were approximately 50% higher during heated‐leg exercise than control‐leg exercise; however, no changes in conduit arterial diameter were observed (Figure 7). According to the Hagen–Poiseuille law, vascular conductance (the inverse of resistance) is predominantly determined by vessel diameter and blood viscosity. It is possible that vasodilatation occurred downstream in the small arteries and resistance arterioles (i.e. microcirculation), thereby contributing to the increases in blood velocity and flow in both age groups (Koch Esteves et al., 2021). During passive hyperthermia in young adults, myriad potential thermosensitive mechanisms have been shown to positively impact blood velocity and thus blood flow. Thermal stimuli could activate intravascular signalling transduction mechanisms (Gifford et al., 2014; Kellogg et al., 1999; Laughlin et al., 2008; Minson et al., 2001; Paniagua et al., 2001) and/or stimulate the release of vasoactive molecules from erythrocytes such as ATP (Kalsi & González‐Alonso, 2012; Kalsi et al., 2017; Pearson et al., 2011), which in turn induce vasodilatation of the resistance vessels. Additionally, reductions in blood viscosity and frictional resistance (Çinar et al., 2001; Lim et al., 2010; Shin et al., 2004; Snyder, 1971) in conjunction with increases in red blood cell deformability and dispersion (Çinar et al., 2001; Manteuffel‐Szoege, 1960, 1969; Pinho et al., 2016) may also play a role in the observed hyperthermia‐induced hyperaemia. However, ageing has been found to negatively impact these thermosensitive and rheological mechanisms, evoking increases in blood viscosity (Carallo et al., 2011; Simmonds et al., 2013), reductions in red blood cell deformability (Simmonds et al., 2013) and an impaired response to various vasodilators (Holowatz & Kenney, 2010; Mortensen et al., 2012; Wray & Richardson, 2015; Hearon Jr & Dinenno, 2016). Regular engagement in physical activity has been shown to preserve these mechanisms (Ernst, 1987; Groot et al., 2016; Mortensen et al., 2012; Simmonds et al., 2013) and thus may explain why the present elderly and young cohorts showed comparable magnitudes of hyperaemia. Nonetheless, if these thermosensitive vascular mechanisms and blood rheological properties do indeed worsen with age, they could at least in part elucidate why the present elderly cohort exhibited a lower blood velocity following heating, knee‐extensor exercise and their combination.

The present elderly cohort also displayed a larger diameter across all measured conduit arteries which was sufficient to compensate for the lower blood velocity (Figure 4). An age‐related increase in arterial diameter is well‐established in the literature (Gonzales et al., 2009; Hirata et al., 2006; Kawasaki et al., 1987; Sandgren et al., 1999), with the aortic diameter increasing by as much as 24% between the ages of 25 and 70 years (Sonesson et al., 1993). These increases are generally associated with an increase in arterial stiffness (Kawasaki et al., 1987; Sonesson et al., 1993) and are therefore indicative of an impaired endothelial‐dependent vasodilator function (Anderson, 2006). However, Hickson et al. (2010) postulated that an increase in (aorta) diameter without a proportional increase in arterial wall thickness may be an adaptation to offset the age‐related increase in arterial stiffness. In this construct, one could speculate that the increased femoral artery diameter in the elderly cohort is an adaptive response to maintain flow rate as blood velocity is attenuated, a hypothesis that warrants further investigation.

### 4.3 Experimental considerations

There are several methodological considerations in this study. First, the present experimental design included two fixed absolute exercise workloads – 6 and 12 W – for all participants. Absolute, low‐intensity workloads were selected as the approach allowed for the direct comparison of hyperaemia between the control and hyperthermic conditions, and between old and young cohorts. Whilst utilising relative workloads would have merit, it is important to acknowledge that the overtime power output variability per kick is large during single‐leg knee‐extensor exercise (González‐Alonso et al., 2000). Thus, using relative workloads—especially if differences in workloads were less than 6 W would have introduced additional variability between protocols and cohorts. Although comparing absolute workloads can pose some limitations (Donato et al., 2006), the present observation that exercise hyperaemia was not different in the elderly and young cohorts suggests that the likely higher relative intensity in the elderly did not affect the outcomes of the study. Second, arterial leg blood velocity, flow and tissue oxygen saturation did not initially increase as rapidly, and to the same magnitude, as local skin temperature. Figures 4 and 5 clearly show that whole‐leg hyperaemia lagged in comparison to the rapid increase in mean leg skin temperature. This initial uncoupling of temperature and haemodynamics was likely due to internal tissue temperature not being measured; thus, it could not be included in the calculation of mean leg temperature and help establish the impact of whole‐leg tissue hyperthermia on limb haemodynamics. When using hot‐water‐perfused garments, as in this study, deep tissue temperature takes longer to increase because conductive heat transfer relies on a positive temperature gradient between the heated skin and the cooler deep tissues. This likely explains why the present correlations were not as strong as those reported in our previous study, which had estimates of regional leg hyperthermia (Koch Esteves et al., 2021). Notwithstanding this limitation, our data lend further support to the notion that internal temperature and concomitant increases in deep tissue blood flow contribute to the observed hyperthermia‐induced limb hyperaemia (Chiesa et al., 2016; Heinonen et al., 2011; Koch Esteves et al., 2021; Pearson et al., 2011). Third, participants were considered *trained* due to their regular participation in structured exercise, which was assessed via a self‐completed questionnaire as opposed to an objective quantification of exercise capacity (e.g. a maximal oxygen consumption test). Although this modality of training status assessment is liable to self‐desirability bias (Nederhof, 1985), we are confident that our participants are indeed trained – particularly our elderly cohort – as their leg muscle mass was comparable to the young cohort. This differs from non‐trained elderly individuals where leg muscle mass is decreased by ∼16% when compared to young 25‐year‐old adults (Naruse et al., 2023). Consequently, the present elderly cohort was classified as *trained* as they did not present characteristics of normal age‐related sarcopenia. Whilst this study attempted to isolate the effect of age on the haemodynamic responses to single‐limb hyperthermia and exercise, rather than the commonly associated age‐related reductions in physical activity, the present findings should not be extrapolated to aged sedentary individuals who will exhibit significant sarcopenia. Last, although lower limb hyperthermia increased blood flow in all major vessels in both the elderly and young groups, the magnitude of increase in blood flow was greater in SFA than PFA. This could be due to the thermal intervention inducing a smaller increase in deep tissue temperature, which is typically warmer than the superficial tissues (Savard et al., 1988), thus producing a weaker thermal stimulus for vasodilatation and/or haemorheological changes. Moreover, measurements of PFA blood flow are influenced by the individual vessel anatomy (Tomaszewski et al., 2017), making it more difficult to obtain high‐quality images compared to the other main leg conduit arteries (Hussain, 1997; Koch Esteves & Chiesa, 2021). Based on PFA blood flow estimates from the measurements of CFA and SFA blood flow, it appears that the magnitude of increase in PFA blood flow was underestimated. Therefore, this limitation does not have a bearing on the conclusions of the study.

### 4.4 Summary

Passive lower limb heating increased leg blood flow and vascular conductance over three‐fold – alongside increases in regional tissue haemoglobin oxygen saturation – which occurred in parallel to increases in local temperature. Moreover, passive leg heating had an additive effect on blood flow during knee‐extensor exercise with the combination of hyperthermia and exercise exhibiting the largest magnitude of functional hyperaemia. Notably, no differences in the functional hyperaemic responses were observed between the healthy, active elderly and the young cohorts despite the larger femoral artery diameter, and lower central haemodynamics and blood velocity in the elderly. These findings reject the idea that age per se compromises local hyperthermia‐induced limb hyperperfusion or small muscle group functional hyperaemia, notwithstanding structural age‐related differences in the vasculature. Further research is warranted to investigate the hyperaemic responses to local limb heating and larger muscle mass exercise – such as walking – in elderly active and sedentary participants to establish the safety and effectiveness of combined local heating and exercise in enhancing circulatory function and vascular health.

## AUTHOR CONTRIBUTIONS

This study was performed at Brunel University London, Uxbridge, UK. Nuno Koch Esteves and José González‐Alonso conceived and designed the research. Nuno Koch Esteves and José González‐Alonso acquired the data. Nuno Koch Esteves analysed the data. Nuno Koch Esteves, Ashraf W. Khir and José González‐Alonso interpreted the data. All authors revised the manuscript and provided intellectual feedback. All authors have read and approved the final version of this manuscript and agree to be accountable for all aspects of the work in ensuring that questions related to the accuracy or integrity of any part of the work are appropriately investigated and resolved. All persons designated as authors qualify for authorship, and all those who qualify for authorship are listed.

## CONFLICT OF INTEREST

The authors declare no conflicts of interest.

## FUNDING INFORMATION

This study was completed without external funding.

## Data Availability

The raw, unidentified data collected throughout this study will be made available via Brunel Figshare, an online data repository database.
